# Effectiveness of continuous venovenous hemodiafiltration using a polymethylmethacrylate membrane hemofilter judging from a multiplex suspension array system in septic shock patients

**DOI:** 10.1186/cc9538

**Published:** 2011-03-11

**Authors:** Y Sakamoto, T Miyasho, N Kutsukata, T Ito, T Iwamura, A Nakashima, M Yahata, K Mashiko, H Yokota, T Obata

**Affiliations:** 1 Saga University Hospital, Saga City, Japan; 2 Rakuno Gakuen University, Sapporo, Japan; 3 Chiba Hokusou Hospital, Nippon Medical School, Inzai, Japan; 4 Nippon Medical School, Tokyo, Japan; 5 Microbial Chemistry Research Foundation, Tokyo, Japan

## Introduction

Septic shock is a condition associated with diffuse coagulopathy and multiple organ failure, and frequently ends in death. The effectiveness of continuous venovenous hemodiafiltration using a polymethylmethacrylate membrane hemofilter (CVVHDF using PMMA) for critically ill patients has also been reported. This treatment was showed as cytokine adsorption therapy, but there are not so many reports in the world.

## Methods

We treated 16 septic shock patients by CVVHDF using PMMA. The patients were checked for 17 kinds of cytokines (IL-1, IL-2, IL-4, IL-5, IL-6, IL-7, IL-8, IL-10, IL-12, IL-13, IL-17, TNFα, G-CSF, GM-CSF, IFNγ, MIP-1, MCP-1/MCAF) using a multiplex suspension array system. We also checked the PMMA column.

## Results

The average APACHE II score and the average sepsis-related organ failure assessment (SOFA) score were 25.8 ± 12.5 and 10.1 ± 3.3 (Bio-Plex™). The survival rate was 83.3%. One day after treatment by CVVHDF using PMMA, IL-1β (*P *= 0.0473), IL-4 (*P *= 0.0206), IL-5 (*P *= 0.0436), IL-7 (*P *= 0.0061), IL-12 (*P *= 0.0049), IL-13 (*P *= 0.0150), IL-17 (*P *= 0.0036), IFNγ (*P *= 0.0308) and TNFα (*P *= 0.0208) were significantly decreased. And 3 days after this treatment, IL-6 (*P *= 0.0498), GC-SF (*P *= 0.0144) and MCP (*P *= 0.0134) were significantly decreased.

## Conclusions

Therapies aimed at blood purification, such as CVVHDF, continuous hemofiltration (CVVHF) and plasma exchange, have been reported to be effective for the removal of inflammatory cytokines and various mediators. Few reports have shown the influence of the column used for CVVHDF on the removal efficiency of the above-mentioned factors, although several columns have been used in CVVHDF. CVVHDF using PMMA has been reported to be effective for cytokine removal. Our findings suggest that many cytokines were decreased after CVVHDF using PMMA treatment. On the other hand, we checked adsorption of many sepsis-related factors on a PMMA column.
